# The crystal structures of Fe-bearing MgCO_3_ *sp* ^2^- and *sp* ^3^-carbonates at 98 GPa from single-crystal X-ray diffraction using synchrotron radiation

**DOI:** 10.1107/S2056989020005411

**Published:** 2020-04-21

**Authors:** Stella Chariton, Maxim Bykov, Elena Bykova, Egor Koemets, Timofey Fedotenko, Björn Winkler, Michael Hanfland, Vitali B. Prakapenka, Eran Greenberg, Catherine McCammon, Leonid Dubrovinsky

**Affiliations:** a Bayerisches Geoinstitut, University of Bayreuth, 95440 Bayreuth, Germany; b Deutsches Elektronen-Synchrotron (DESY), 22607 Hamburg, Germany; c Material Physics and Technology at Extreme Conditions, Laboratory of Crystallography, University of Bayreuth, 95440 Bayreuth, Germany; d Institute of Geosciences, Goethe University, 60438 Frankfurt am Main, Germany; e European Synchrotron Radiation Facility, BP 220, 38043 Grenoble Cedex, France; f GeoSoilEnviroCARS, University of Chicago, 60637 Chicago, Illinois, USA

**Keywords:** carbonates, magnesite-II, *sp*^3^-carbonates, *sp*^2^-carbonates, crystal structure, high-pressure single-crystal X-ray diffraction

## Abstract

The crystal structure of MgCO_3_-II has long been discussed in the literature where DFT-based model calculations predict a pressure-induced transition of the carbon atom from the *sp* ^2^ to the *sp* ^3^ type of bonding. We have now determined the crystal structure of iron-bearing MgCO_3_-II based on single-crystal X-ray diffraction measurements using synchrotron radiation. We laser-heated a synthetic (Mg_0.85_Fe_0.15_)CO_3_ single crystal at 2500 K and 98 GPa and observed the formation of a monoclinic phase with composition (Mg_2.53_Fe_0.47_)C_3_O_9_ in the space group *C*2/*m* that contains tetra­hedrally coordinated carbon, where CO_4_^4−^ tetra­hedra are linked by corner-sharing oxygen atoms to form three-membered C_3_O_9_^6−^ ring anions. The crystal structure of (Mg_0.85_Fe_0.15_)CO_3_ (magnesium iron carbonate) at 98 GPa and 300 K is reported here as well. In comparison with previous structure-prediction calculations and powder X-ray diffraction data, our structural data provide reliable information from experiments regarding atomic positions, bond lengths, and bond angles.

## Abstract 2

We report the phase transition of Mg_0.85_Fe_0.15_CO_3_ *sp* ^2^- to Mg_2.53_Fe_0.47_C_3_O_9_ *sp* ^3^-carbonate (MgCO_3_–II phase) at 98 GPa and describe their crystal structures by means of single-crystal X-ray diffraction experiments in laser-heated diamond anvil cells.

## Chemical context   

Carbonates and their high-pressure behaviour have attracted significant inter­est because of their potential role as carbon-bearing phases in the deep Earth. Recent discoveries of novel compounds that contain tetra­hedral CO_4_^4−^ units (*e.g.*, Merlini *et al.*, 2015 ▸; Cerantola *et al.*, 2017 ▸) increase the relevance of such studies, as the new high-pressure phases may be stable at conditions prevalent in the deep part of Earth’s lower mantle. In addition, theoretical modelling predictions imply potential structural analogues of CO_4_^4−^-bearing carbonates and silicates, and thus carbonates with tetra­hedrally coordinated carbon may be important to understanding the complex geochemistry of Earth’s mantle.

Carbonates with tetra­hedrally coordinated carbon are not well characterized, despite their potential significance, as structural studies have to be carried out under high-pressure conditions and are therefore challenging. A reliable structural characterization is, however, a prerequisite for determining phase stabilities and to understand, for example, why the *p*,*T*-phase diagram of MgCO_3_ is relatively simple compared to the dense phase diagram of CaCO_3_ (see summary in Bayarjargal *et al.*, 2018 ▸).

It is generally accepted that magnesite (MgCO_3_) transforms to MgCO_3_-II at 80–115 GPa (Isshiki *et al.*, 2004 ▸; Boulard *et al.*, 2011 ▸,2015 ▸; Maeda *et al.*, 2017 ▸). Models based on density functional theory (DFT) (Oganov *et al.*, 2008 ▸) and inter­pretation of X-ray diffraction data and IR spectra imply that MgCO_3_-II contains carbon in a tetra­hedral coordination (Boulard *et al.* 2011 ▸, 2015 ▸). While structure-prediction techniques are undoubtedly useful for preliminary surveys of phase stabilities, they provide a range of possible new phases, derived under constraints such as unit-cell contents. Powder diffraction data obtained at pressures around 100 GPa generally do not yield accurate structure determinations, and typically do not allow unambiguous assignment of the space group or site occupancies. In contrast, single-crystal X-ray diffraction is a powerful and unique tool that can provide accurate structure refinements under these conditions (Boffa Ballaran *et al.*, 2013 ▸). Well-established statistical parameters allow an assessment of the reliability of the structural model. Other carbonate structures with tetra­hedral CO_4_^4−^ units at extreme conditions have previously been reported using this method, such as the novel phases Fe_4_C_3_O_12_ in space group *R*3*c*, (Mg,Fe)_4_C_4_O_13_ in *C*2/*c* (Merlini *et al.*, 2015 ▸; Cerantola *et al.*, 2017 ▸) and Ca(Fe,Mg)_2_C_3_O_9_ in *Pnma (*Merlini *et al.*, 2017 ▸). These results lead to two conclusions. Firstly, the stability fields of carbonates strongly depend on their composition. Secondly, CO_4_^4−^ units have the ability to form polymeric networks, and thus are potential analogues to silicates.

## Structural commentary   

**Figure 1:**
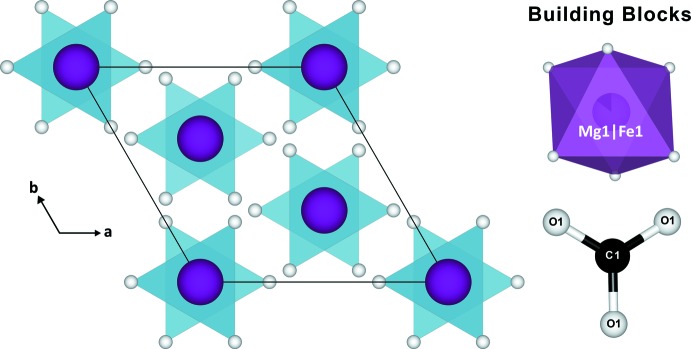
Crystal structure of (Mg_0.85_Fe_0.15_)CO_3_ at 98 GPa and prior to laser-heating shown in a projection along the *c* axis. The building blocks of the unit cell appear on the right. Here, iron occupies the same sites as the magnesium atoms.

Under ambient conditions (Mg_0.85_Fe_0.15_)CO_3_ crystallizes in the calcite-type structure in space group *R* 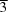 *c*. Iron and magnesium share the same crystallographic site (Wyckoff position 6*b*; site symmetry 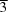.) and are coordinated by six oxygen atoms, while the CO_3_^2−^ units form planar equilateral triangles with point-group symmetry 32 (*e.g.* Lavina *et al.*, 2010 ▸). After compression to 98 (2) GPa at ambient temperature, X-ray diffraction data of (Mg_0.85_Fe_0.15_)CO_3_ can still be indexed in the *R* 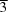 *c* space group (Fig. 1 ▸, Table 1 ▸). However, the unit-cell volume is decreased by nearly 32% compared to ambient conditions. This result challenges a recent suggestion based on DFT-based calculations that predicted a structural transformation of MgCO_3_ to a triclinic phase at 85–101 GPa and 300 K (Pickard & Needs, 2015 ▸). At 98 GPa, the C—O bond length [1.195 (8) Å] has decreased only by ∼7% compared to the structure at ambient conditions, thus reflecting the highly incompressible nature of the CO_3_^2−^ units. On the other hand, the (Mg/Fe)—O bonds [1.855 (5) Å at 98 GPa] display a much more compressible behavior (∼12% bond-length and ∼32% octa­hedra-volume shrinkage compared to ambient conditions). On a last note, it is well known that rhombohedral carbonates can be described as a distortion of the NaCl (B1) structure. Previously, the *t* parameter, 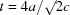, where *a* and *c* are the lattice parameters) has been used to evaluate the degree of distortion (Gao *et al.*, 2014 ▸). We observed that at 98 GPa and 300 K, *t* ≃1 for (Mg_0.85_Fe_0.15_)CO_3_, which means that at the conditions of our experiment the (Mg/Fe) cations and the CO_3_^2−^ anions are arranged in the manner of a nearly ideal NaCl (B1) structure.

**Figure 2:**
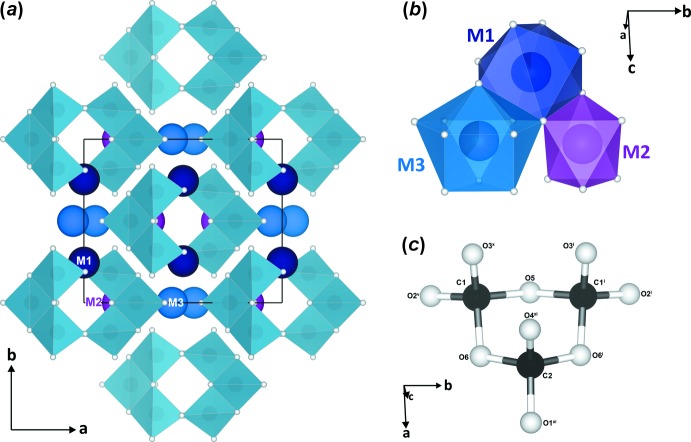
(*a*) The crystal structure of (Mg_2.53_Fe_0.47_)C_3_O_9_ according to this study, in a projection along the *c* axis; CO_4_ tetra­hedra are given in the polyhedral representation. (*b*) The three cation sites that host Mg/Fe atoms and their respective polyhedra. (*c*) C_3_O_9_^6−^ ring anions are formed from three edge-sharing CO_4_ tetra­hedra. Atomic positions are shaded according to colours in (*b*) and oxygen atoms appear as small white spheres. [Symmetry codes: (i) *x*, −*y*, *z*; (v) −*x* + 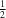, −*y* + 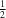, −*z* + 1; (*x*) −*x*, *y*, −*z* + 1; (xi) *x*, *y*, *z* + 1.]

After annealing at 2500 K and 98 GPa, we observed a phase transition to a polymorph in which carbon is tetra­hedrally coordinated by oxygen. The newly formed phase with chemical formula (Mg_2.53_Fe_0.47_)C_3_O_9_ (as determined from structural refinements, see below) has monoclinic symmetry, and the diffraction pattern indicates space group *C*2/*m* (Fig. 2 ▸, Table 1 ▸). We identify this phase as the MgCO_3_-II structure that was previously predicted (Oganov *et al.*, 2008 ▸; Boulard *et al.*, 2015 ▸). In contrast to previous studies, we provide an accurate structure solution and refinement based on single crystal X-ray diffraction data. The structure consists of three-membered C_3_O_9_^6−^ rings formed by corner-sharing CO_4_ tetra­hedra (Fig. 2 ▸ *c*) that alternate with [Fe,Mg]O_*x*_ polyhedra (*x* = 6–8) perpendicular to the *b* axis. We can distinguish three crystallographic cation positions (Fig. 2 ▸ *b*):

(1) The *M*1 site is located on a twofold rotation axis (Wyckoff position 4*g*) and is occupied by Mg and Fe in a 0.917 (17):0.083 (17) ratio. This site is surrounded by eight oxygen atoms forming a distorted square anti­prism (dark blue); (2) The *M*3 site is situated on a mirror plane (4 *i*) in a 0.61 (2):0.39 (2) Mg:Fe ratio and a coordination number of 10 (blue; can be described as half cubocta­hedra merged through hexa­gonal-based faces with hexa­gonal pyramids); (3) *M*2 is likewise situated on a mirror plane (4 *i*) and is fully occupied by Mg in [MgO_6_] octa­hedra (magenta). The maximum and minimum bond lengths of each cation site from its neighbouring oxygen atoms are shown in Table 2 ▸. At 98 GPa the C—O bond lengths of the two different CO_4_^4−^ carbonate groups [C1 is located on a general site (8 *j*) and C2 on a mirror plane (4 *i*) vary from 1.287 (18)–1.409 (13) Å and the C—O—C inter-tetra­hedral angle is ∼112°.

From all proposed structural models for MgCO_3_-II over the last two decades, only one appears to successfully match the structure model that we report here. On the basis of powder X-ray diffraction (PXRD) experiments and variable-cell simulations, Oganov *et al.* (2008 ▸) suggested several energet­ically favourable structural models for MgCO_3_-II, one of which is in space group *C*2/*m*. While our structural solution and refinement from the experimental data is clearly similar to the theoretical predictions by Oganov *et al.* (2008 ▸), the different composition of the materials and the small differences in the structural parameters required us to check additionally whether theoretical calculations with our model as the starting one would lead to the same result as that reported by Oganov *et al.* (2008 ▸). We performed such a test and confirm that our results and those of Oganov *et al.* (2008 ▸) are the same within the accuracy of the methods. More concretely, we performed DFT-based model calculations using the plane wave/pseudopotential *CASTEP* package (Clark *et al.*, 2005 ▸). Pseudopotentials were generated ‘on the fly’ using the parameters provided with the *CASTEP* distribution. These pseudopotentials have been tested extensively for accuracy and transferability (Lejaeghere *et al.*, 2016 ▸). The pseudopotentials were employed in conjunction with plane waves up to a kinetic energy cutoff of 1020 eV. The calculations were carried out with the PBE exchange–correlation function (Perdew *et al.*, 1996 ▸). For simplicity, we assumed that all three *M*1, *M*2 and *M*3 positions are fully occupied by Mg^2+^. The calculations revealed that the energies of our structural model and that of Oganov *et al.* (2008 ▸) are indeed, identical. The DFT calculations gave C—O distances in good agreement with experimental data. Each carbon atom is coordinated by two oxygen atoms that are each shared with another tetra­hedrally coord­inated carbon, and two that are not shared. The C—O distances for the latter are significantly shorter [1.29 Å < *d*(C—O) < 1.32 Å] than the former [1.33 Å < (C—O) < 1.41 Å]. A Mulliken bond-population analysis shows that for the long C—O bonds there is a significant bond population of ∼0.5 e^−^ Å^−3^. This is less than the value for the short bonds, where the bond population is ∼0.9 e^−^ Å^−3^, but this still is a predominantly covalent bond, and justifies the description as a tetra­hedrally coordinated carbon atom. The formation of (C_3_O_9_)^6−^ carbonate rings was previously observed in Ca(Fe,Mg)C_3_O_9_ (dolomite-IV) after laser heating of Ca(Fe,Mg)CO_3_ at 115 GPa (Merlini *et al.*, 2017 ▸). However, dolomite-IV is topologically different from the MgCO_3_-II structure that we report here. Unlike (Mg_2.53_Fe_0.47_)C_3_O_9_, Ca(Fe,Mg)C_3_O_9_ crystallizes in the ortho­rhom­bic system (space group *Pnma*), thus highlighting the significance of the metal cations that are present in the carbonate.

**Figure 3:**
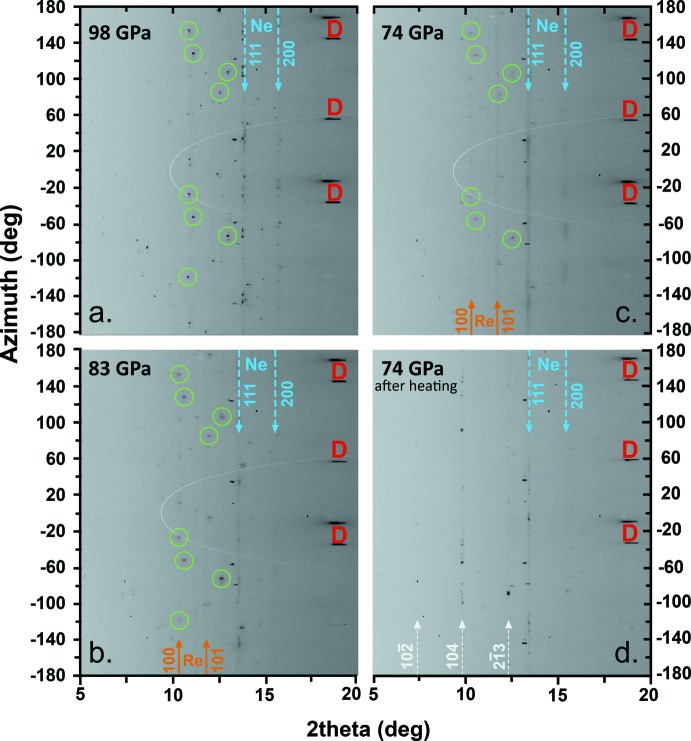
Unrolled X-ray diffraction images collected at room temperature (λ = 0.411 Å). (*a*) Sharp and intense reflections of (Mg_2.53_Fe_0.47_)C_3_O_9_ appear after laser-heating of the starting material at 98 GPa and 2500 K. (*b*) The crystal phase gradually deteriorates during decompression and (*c*) nearly disappears at ∼74 GPa. (*d*) Consequent laser-heating treatment results in the formation of the initial carbonate structure. Green circles mark a few of the characteristic reflections of (Mg_2.53_Fe_0.47_)C_3_O_9_, the position of Ne reflections and in some cases Re reflections are marked with blue and orange arrows, respectively. The 2*θ* positions of three characteristic carbonate (*R* 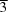 *c*) reflections are indicated with white arrows. Diamond reflections are marked in red.

Upon decompression at ambient temperature, (Mg_2.53_Fe_0.47_)C_3_O_9_ reflections become broad and weak, and almost disappear at ∼74 GPa (Fig. 3 ▸ *a*–*c*). This may be an indication of either amorphization or sluggish back-transformation to a carbonate with trigonal symmetry. Anti­cipating that further heating would aid recrystallization, we laser-heated the sample at 74 GPa and 2000 (150) K for a few seconds. Wide images collected on the temperature-quenched sample indicated the formation of the calcite structure-type carbonate (Fig. 3 ▸ *d*).

## Synthesis and crystallization   

Magnesium carbonate crystals with 15(±4) mol% Fe were grown following the procedure reported by Chariton *et al.* (2020 ▸). The composition of the starting material was determined by single-crystal X-ray diffraction under ambient conditions as (Mg_0.85_Fe_0.15_)CO_3_. A single crystal of ∼7 µm size in all dimensions was loaded inside the sample chamber of a BX90-type diamond anvil cell equipped with bevelled Boehler–Almax type diamonds (culet diameter 80 mm). Rhenium and neon were used as the gasket material and pressure-transmitting medium, respectively. The pressure was determined using the equation of state (EoS) of solid Ne (Fei *et al.*, 2007 ▸). First, the sample was compressed up to 98 GPa and a single-crystal collection took place at 300 K. Consequently, the same crystal was laser-heated from both sides up to 2500 (150) K for a few seconds and then quenched to room temperature. Finally, we performed a 5×5 grid of still-image collection with a 2 µm step and 1 s exposure time around the center of the sample. This strategy was used to locate the most heated area of the crystal and the best spot to collect single-crystal X-ray diffraction patterns during rotation of the cell. Single-crystal data collection was performed as a series of *ω* scans over the range ±35° with a step of 0.5°.

## Refinement   

Details of the data collection, structure solution and refinement are summarized in Table 1 ▸. In the case of the (Mg_0.85_Fe_0.15_)CO_3_ dataset collected at 98 GPa, the limited number of available reflections required us to fix the Fe content according to our ambient condition estimates (see also "Synthesis and Crystallization" section). On the other hand, during the structure refinements of (Mg_2.53_Fe_0.47_)C_3_O_9_ all three cation sites (i.e. *M*1, *M*2 and *M*3) were tested for their ability to host Fe by refining the site occupancies. As described above, only the *M*1 and *M*3 sites were eventually found to accommodate ∼16(±3) mol % Fe in total. Note that the resulting 5.38 Mg:Fe ratio of (Mg_2.53_Fe_0.47_)C_3_O_9_ is almost identical to the starting 5.67 Mg:Fe ratio of (Mg_0.85_Fe_0.15_)CO_3_ within the accuracy of our method. Therefore, it is safe to conclude that nearly none or only a negligible amount of Fe was lost during the observed phase transition. The crystal structure of (Mg_2.53_Fe_0.47_)C_3_O_9_ solved at 98 GPa was used for the structure refinements of the data of the same phase collected during decompression. Due to the limited angular range caused by the laser-heated DAC, the resolution of the data set was not sufficient to refine the anisotropic displacement parameters. Therefore, all atoms were refined with the isotropic approximation.

## Supplementary Material

Crystal structure: contains datablock(s) MgCO3-II_98GPa, MgCO3_98GPa. DOI: 10.1107/S2056989020005411/wm5543sup1.cif

CCDC references: 1998018, 1998019

Additional supporting information:  crystallographic information; 3D view; checkCIF report

## Figures and Tables

**Table 1:** Experimental details

	MgCO_3_-II at 98 GPa	MgCO_3_ at 98 GPa
Crystal data		
Chemical formula	3[(Mg_0.85_Fe_0.15_)CO_3_]	(Mg_0.85_Fe_0.15_)CO_3_
*M*_r_	265.6	89
Crystal system, space group	Monoclinic, *C*2/*m*	Trigonal, *R* 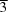 *c*
Temperature (K)	293	293
*a*, *b*, *c* (Å)	8.238 (3), 6.5774 (12), 6.974 (5)	4.281 (7), 4.281 (7), 12.12 (2)
α, β, γ (°)	90, 104.40 (6), 90	90, 90, 120
*V* (Å^3^)	366.0 (3)	192.3 (5)
*Z*	4	6
Radiation type	Synchrotron, λ = 0.41107 Å	Synchrotron, λ = 0.2952 Å
μ (mm^−1^)	0.58	0.25
Crystal size (mm)	0.01 × 0.01 × 0.01	0.01 × 0.01 × 0.01

Data collection		
Diffractometer	ID15b @ ESRF	13IDD @ APS (GSECARS)
Absorption correction	Multi-scan (*CrysAlis PRO*; Rigaku OD, 2019 ▸)	Multi-scan (*CrysAlis PRO*; Rigaku OD, 2019 ▸)
*T*_min_, *T*_max_	0.104, 1	0.95, 1
No. of measured, independent and observed [*I* > 3σ(*I*)] reflections	522, 298, 211	176, 60, 33
*R*_int_	0.020	0.053
(sin θ/λ)_max_ (Å^−1^)	0.860	0.900

Refinement		
*R*[*F* ^2^ > 2σ(*F* ^2^)], *wR*(*F* ^2^), *S*	0.084, 0.095, 3.21	0.100, 0.084, 2.89
No. of reflections	298	60
No. of parameters	39	5
Δρ_max_, Δρ_min_ (e Å^−3^)	1.76, −1.21	0.66, −0.50

Computer programs: *CrysAlis PRO* (Rigaku OD, 2019 ▸), *SUPERFLIP* (Palatinus & Chapuis, 2007 ▸), *JANA2006* (Petříček *et al.*, 2014 ▸), *VESTA* (Momma & Izumi, 2011 ▸) and *publCIF* (Westrip, 2010 ▸).

**Table 2:** Geometric parameters of (Mg_2.53_Fe_0.47_)C_3_O_9_ at 98 GPa

Group	Maximal bond length (Å)	Minimal bond length (Å)	Polyhedron volume (Å^3^)	Distortion index*^*a*^*
CO_4_ (C1—O)	1.409 (19)	1.287 (18)	1.25	0.045
CO_4_ (C2—O)	1.38 (3)	1.29 (4)	1.25	0.022
*M*2O_6_ *^*b*^*	1.87 (3)	1.813 (10)	7.78	0.010
*M*1O_8_ *^*c*^*	2.039 (13)	1.908 (14)	13.24	0.020
*M*3O_8_ *^*d*^*	2.358 (14)*^*e*^*	1.828 (19)	14.59	0.068

Notes: (*a*) as defined in Baur (1974 ▸); (*b*) Mg:Fe ratio for *M* = 1:0; (*c*) Mg:Fe ratio for *M* = 0.917 (17):0.083 (17); (*d*) Mg:Fe ratio for *M* = 0.61 (2):0.39 (2); (*e*) alternatively, for CN = 10 the maximal distance is 2.451 (14) Å, the polyhedral volume is 20.58 Å^3^ and the distortion index is 0.080.

## supplementary crystallographic information

### Magnesium(II) iron(II) carbonate (MgCO3-II_98GPa) Crystal data

**Table:**

3[(Mg_0.85_Fe_0.15_)CO_3_]	*F*(000) = 530
*M**_r_* = 265.6	*D*_x_ = 4.861 Mg m^−^^3^
Monoclinic, *C*2/*m*	Synchrotron radiation, λ = 0.41107 Å
Hall symbol: -C 2y	Cell parameters from 146 reflections
*a* = 8.238 (3) Å	θ = 2.3–19.0°
*b* = 6.5774 (12) Å	µ = 0.58 mm^−^^1^
*c* = 6.974 (5) Å	*T* = 293 K
β = 104.40 (6)°	Irregular, colourless
*V* = 366.0 (3) Å^3^	0.01 × 0.01 × 0.01 mm
*Z* = 4	

### Magnesium(II) iron(II) carbonate (MgCO3-II_98GPa) Data collection

**Table:**

ID15b @ ESRF diffractometer	298 independent reflections
Radiation source: synchrotron	211 reflections with *I* > 3σ(*I*)
Synchrotron monochromator	*R*_int_ = 0.020
ω scans	θ_max_ = 20.7°, θ_min_ = 2.3°
Absorption correction: multi-scan (CrysAlisPro; Rigaku OD, 2019)	*h* = −11→12
*T*_min_ = 0.104, *T*_max_ = 1	*k* = −8→8
522 measured reflections	*l* = −7→9

### Magnesium(II) iron(II) carbonate (MgCO3-II_98GPa) Refinement

**Table:**

Refinement on *F*	0 restraints
Least-squares matrix: full	5 constraints
*R*[*F*^2^ > 2σ(*F*^2^)] = 0.084	Weighting scheme based on measured s.u.'s *w* = 1/(σ^2^(*F*) + 0.000144*F*^2^)
*wR*(*F*^2^) = 0.095	(Δ/σ)_max_ = 0.001
*S* = 3.21	Δρ_max_ = 1.76 e Å^−^^3^
298 reflections	Δρ_min_ = −1.21 e Å^−^^3^
39 parameters	

### Magnesium(II) iron(II) carbonate (MgCO3-II_98GPa) Fractional atomic coordinates and isotropic or equivalent isotropic displacement parameters (Å^2^)

**Table:**

	*x*	*y*	*z*	*U*_iso_*/*U*_eq_	Occ. (<1)
Mg3	0.4441 (6)	0	0.6503 (9)	0.0177 (11)*	0.61 (2)
Fe3	0.4441 (6)	0	0.6503 (9)	0.0177 (11)*	0.39 (2)
Mg2	0.1712 (7)	0	0.3146 (12)	0.0086 (11)*	
Mg1	0	0.2457 (6)	0	0.0117 (13)*	0.917 (17)
Fe1	0	0.2457 (6)	0	0.0117 (13)*	0.083 (17)
O4	0.1395 (17)	0	0.044 (3)	0.020 (2)*	
O6	0.2736 (13)	0.1662 (9)	0.847 (2)	0.0179 (17)*	
O2	0.3442 (12)	0.1683 (9)	0.4218 (18)	0.0157 (15)*	
O1	0.4097 (18)	0	0.105 (3)	0.021 (2)*	
O5	0.1487 (16)	0	0.575 (3)	0.016 (2)*	
O3	0.0062 (12)	0.1898 (9)	0.2702 (19)	0.0159 (17)*	
C1	0.1347 (19)	0.1774 (13)	0.683 (3)	0.017 (2)*	
C2	0.265 (3)	0	0.964 (4)	0.024 (3)*	

### Magnesium(II) iron(II) carbonate (MgCO3-II_98GPa) Geometric parameters (Å, º)

**Table:**

Mg3—O6	2.451 (14)	Mg2—O3^i^	1.814 (9)
Mg3—O6^i^	2.451 (14)	Mg1—O4	1.962 (8)
Mg3—O2	1.947 (11)	Mg1—O4^vi^	1.962 (8)
Mg3—O2^ii^	2.226 (11)	Mg1—O6^v^	1.991 (10)
Mg3—O2^iii^	2.226 (11)	Mg1—O6^vii^	1.991 (10)
Mg3—O2^i^	1.947 (11)	Mg1—O1^viii^	2.039 (12)
Mg3—O1^ii^	1.829 (18)	Mg1—O1^ix^	2.039 (12)
Mg3—O5	2.359 (14)	Mg1—O3	1.908 (13)
Mg3—O3^iv^	2.127 (7)	Mg1—O3^vi^	1.908 (13)
Mg3—O3^v^	2.127 (7)	Fe1—O4	1.962 (8)
Fe3—O6	2.451 (14)	Fe1—O4^vi^	1.962 (8)
Fe3—O6^i^	2.451 (14)	Fe1—O6^v^	1.991 (10)
Fe3—O2	1.947 (11)	Fe1—O6^vii^	1.991 (10)
Fe3—O2^ii^	2.226 (11)	Fe1—O1^viii^	2.039 (12)
Fe3—O2^iii^	2.226 (11)	Fe1—O1^ix^	2.039 (12)
Fe3—O2^i^	1.947 (11)	Fe1—O3	1.908 (13)
Fe3—O1^ii^	1.829 (18)	Fe1—O3^vi^	1.908 (13)
Fe3—O5	2.359 (14)	C1—O6	1.403 (19)
Fe3—O3^iv^	2.127 (7)	C1—O2^v^	1.288 (18)
Fe3—O3^v^	2.127 (7)	C1—O5	1.411 (17)
Mg2—O4	1.84 (2)	C1—O3^x^	1.28 (2)
Mg2—O2	1.813 (9)	C2—O4^xi^	1.29 (3)
Mg2—O2^i^	1.813 (9)	C2—O6	1.38 (2)
Mg2—O5	1.87 (2)	C2—O6^i^	1.38 (2)
Mg2—O3	1.814 (9)	C2—O1^xi^	1.34 (3)
			
O6—Mg3—O6^i^	53.0 (3)	O5—Fe3—O3^iv^	100.3 (3)
O6—Mg3—O2	91.1 (4)	O5—Fe3—O3^v^	100.3 (3)
O6—Mg3—O2^ii^	119.7 (3)	O3^iv^—Fe3—O3^v^	147.1 (5)
O6—Mg3—O2^iii^	159.8 (5)	O4—Mg2—O2	108.6 (6)
O6—Mg3—O2^i^	121.8 (4)	O4—Mg2—O2^i^	108.6 (6)
O6—Mg3—O1^ii^	79.3 (6)	O4—Mg2—O5	166.6 (7)
O6—Mg3—O5	54.7 (5)	O4—Mg2—O3	85.1 (6)
O6—Mg3—O3^iv^	112.4 (5)	O4—Mg2—O3^i^	85.1 (6)
O6—Mg3—O3^v^	61.7 (4)	O2—Mg2—O2^i^	75.3 (4)
O6^i^—Mg3—O2	121.8 (4)	O2—Mg2—O5	81.8 (6)
O6^i^—Mg3—O2^ii^	159.8 (5)	O2—Mg2—O3	97.4 (4)
O6^i^—Mg3—O2^iii^	119.7 (3)	O2—Mg2—O3^i^	165.8 (7)
O6^i^—Mg3—O2^i^	91.1 (4)	O2^i^—Mg2—O5	81.8 (6)
O6^i^—Mg3—O1^ii^	79.3 (6)	O2^i^—Mg2—O3	165.8 (7)
O6^i^—Mg3—O5	54.7 (5)	O2^i^—Mg2—O3^i^	97.4 (4)
O6^i^—Mg3—O3^iv^	61.7 (4)	O5—Mg2—O3	85.1 (6)
O6^i^—Mg3—O3^v^	112.4 (5)	O5—Mg2—O3^i^	85.1 (6)
O2—Mg3—O2^ii^	74.2 (4)	O3—Mg2—O3^i^	87.0 (4)
O2—Mg3—O2^iii^	107.0 (5)	O4—Mg1—O4^vi^	69.1 (5)
O2—Mg3—O2^i^	69.3 (4)	O4—Mg1—O6^v^	73.9 (4)
O2—Mg3—O1^ii^	144.2 (3)	O4—Mg1—O6^vii^	139.3 (4)
O2—Mg3—O5	67.3 (5)	O4—Mg1—O1^viii^	150.6 (7)
O2—Mg3—O3^iv^	140.7 (5)	O4—Mg1—O1^ix^	118.7 (6)
O2—Mg3—O3^v^	71.5 (4)	O4—Mg1—O3	79.4 (6)
O2^ii^—Mg3—O2^iii^	59.6 (3)	O4—Mg1—O3^vi^	82.3 (6)
O2^ii^—Mg3—O2^i^	107.0 (5)	O4^vi^—Mg1—O6^v^	139.3 (4)
O2^ii^—Mg3—O1^ii^	80.8 (6)	O4^vi^—Mg1—O6^vii^	73.9 (4)
O2^ii^—Mg3—O5	140.5 (4)	O4^vi^—Mg1—O1^viii^	118.7 (6)
O2^ii^—Mg3—O3^iv^	115.2 (4)	O4^vi^—Mg1—O1^ix^	150.6 (7)
O2^ii^—Mg3—O3^v^	58.1 (4)	O4^vi^—Mg1—O3	82.3 (6)
O2^iii^—Mg3—O2^i^	74.2 (4)	O4^vi^—Mg1—O3^vi^	79.4 (6)
O2^iii^—Mg3—O1^ii^	80.8 (6)	O6^v^—Mg1—O6^vii^	146.1 (3)
O2^iii^—Mg3—O5	140.5 (4)	O6^v^—Mg1—O1^viii^	86.9 (5)
O2^iii^—Mg3—O3^iv^	58.1 (4)	O6^v^—Mg1—O1^ix^	64.9 (5)
O2^iii^—Mg3—O3^v^	115.2 (4)	O6^v^—Mg1—O3	74.6 (5)
O2^i^—Mg3—O1^ii^	144.2 (3)	O6^v^—Mg1—O3^vi^	112.2 (5)
O2^i^—Mg3—O5	67.3 (5)	O6^vii^—Mg1—O1^viii^	64.9 (5)
O2^i^—Mg3—O3^iv^	71.5 (4)	O6^vii^—Mg1—O1^ix^	86.9 (5)
O2^i^—Mg3—O3^v^	140.7 (5)	O6^vii^—Mg1—O3	112.2 (5)
O1^ii^—Mg3—O5	127.7 (8)	O6^vii^—Mg1—O3^vi^	74.6 (5)
O1^ii^—Mg3—O3^iv^	73.6 (3)	O1^viii^—Mg1—O1^ix^	69.7 (7)
O1^ii^—Mg3—O3^v^	73.6 (3)	O1^viii^—Mg1—O3	74.1 (6)
O5—Mg3—O3^iv^	100.3 (3)	O1^viii^—Mg1—O3^vi^	126.1 (6)
O5—Mg3—O3^v^	100.3 (3)	O1^ix^—Mg1—O3	126.1 (6)
O3^iv^—Mg3—O3^v^	147.1 (5)	O1^ix^—Mg1—O3^vi^	74.1 (6)
O6—Fe3—O6^i^	53.0 (3)	O3—Mg1—O3^vi^	157.8 (4)
O6—Fe3—O2	91.1 (4)	O4—Fe1—O4^vi^	69.1 (5)
O6—Fe3—O2^ii^	119.7 (3)	O4—Fe1—O6^v^	73.9 (4)
O6—Fe3—O2^iii^	159.8 (5)	O4—Fe1—O6^vii^	139.3 (4)
O6—Fe3—O2^i^	121.8 (4)	O4—Fe1—O1^viii^	150.6 (7)
O6—Fe3—O1^ii^	79.3 (6)	O4—Fe1—O1^ix^	118.7 (6)
O6—Fe3—O5	54.7 (5)	O4—Fe1—O3	79.4 (6)
O6—Fe3—O3^iv^	112.4 (5)	O4—Fe1—O3^vi^	82.3 (6)
O6—Fe3—O3^v^	61.7 (4)	O4^vi^—Fe1—O6^v^	139.3 (4)
O6^i^—Fe3—O2	121.8 (4)	O4^vi^—Fe1—O6^vii^	73.9 (4)
O6^i^—Fe3—O2^ii^	159.8 (5)	O4^vi^—Fe1—O1^viii^	118.7 (6)
O6^i^—Fe3—O2^iii^	119.7 (3)	O4^vi^—Fe1—O1^ix^	150.6 (7)
O6^i^—Fe3—O2^i^	91.1 (4)	O4^vi^—Fe1—O3	82.3 (6)
O6^i^—Fe3—O1^ii^	79.3 (6)	O4^vi^—Fe1—O3^vi^	79.4 (6)
O6^i^—Fe3—O5	54.7 (5)	O6^v^—Fe1—O6^vii^	146.1 (3)
O6^i^—Fe3—O3^iv^	61.7 (4)	O6^v^—Fe1—O1^viii^	86.9 (5)
O6^i^—Fe3—O3^v^	112.4 (5)	O6^v^—Fe1—O1^ix^	64.9 (5)
O2—Fe3—O2^ii^	74.2 (4)	O6^v^—Fe1—O3	74.6 (5)
O2—Fe3—O2^iii^	107.0 (5)	O6^v^—Fe1—O3^vi^	112.2 (5)
O2—Fe3—O2^i^	69.3 (4)	O6^vii^—Fe1—O1^viii^	64.9 (5)
O2—Fe3—O1^ii^	144.2 (3)	O6^vii^—Fe1—O1^ix^	86.9 (5)
O2—Fe3—O5	67.3 (5)	O6^vii^—Fe1—O3	112.2 (5)
O2—Fe3—O3^iv^	140.7 (5)	O6^vii^—Fe1—O3^vi^	74.6 (5)
O2—Fe3—O3^v^	71.5 (4)	O1^viii^—Fe1—O1^ix^	69.7 (7)
O2^ii^—Fe3—O2^iii^	59.6 (3)	O1^viii^—Fe1—O3	74.1 (6)
O2^ii^—Fe3—O2^i^	107.0 (5)	O1^viii^—Fe1—O3^vi^	126.1 (6)
O2^ii^—Fe3—O1^ii^	80.8 (6)	O1^ix^—Fe1—O3	126.1 (6)
O2^ii^—Fe3—O5	140.5 (4)	O1^ix^—Fe1—O3^vi^	74.1 (6)
O2^ii^—Fe3—O3^iv^	115.2 (4)	O3—Fe1—O3^vi^	157.8 (4)
O2^ii^—Fe3—O3^v^	58.1 (4)	O6—C1—O2^v^	107.8 (11)
O2^iii^—Fe3—O2^i^	74.2 (4)	O6—C1—O5	103.5 (10)
O2^iii^—Fe3—O1^ii^	80.8 (6)	O6—C1—O3^x^	113.7 (17)
O2^iii^—Fe3—O5	140.5 (4)	O2^v^—C1—O5	107.9 (17)
O2^iii^—Fe3—O3^iv^	58.1 (4)	O2^v^—C1—O3^x^	110.6 (10)
O2^iii^—Fe3—O3^v^	115.2 (4)	O5—C1—O3^x^	112.8 (11)
O2^i^—Fe3—O1^ii^	144.2 (3)	O4^xi^—C2—O6	114.7 (12)
O2^i^—Fe3—O5	67.3 (5)	O4^xi^—C2—O6^i^	114.7 (12)
O2^i^—Fe3—O3^iv^	71.5 (4)	O4^xi^—C2—O1^xi^	110 (3)
O2^i^—Fe3—O3^v^	140.7 (5)	O6—C2—O6^i^	105 (2)
O1^ii^—Fe3—O5	127.7 (8)	O6—C2—O1^xi^	105.7 (13)
O1^ii^—Fe3—O3^iv^	73.6 (3)	O6^i^—C2—O1^xi^	105.7 (13)
O1^ii^—Fe3—O3^v^	73.6 (3)		

Symmetry codes: (i) *x*, −*y*, *z*; (ii) −*x*+1, *y*, −*z*+1; (iii) −*x*+1, −*y*, −*z*+1; (iv) −*x*+1/2, *y*−1/2, −*z*+1; (v) −*x*+1/2, −*y*+1/2, −*z*+1; (vi) −*x*, *y*, −*z*; (vii) *x*−1/2, −*y*+1/2, *z*−1; (viii) *x*−1/2, *y*+1/2, *z*; (ix) −*x*+1/2, *y*+1/2, −*z*; (x) −*x*, *y*, −*z*+1; (xi) *x*, *y*, *z*+1.

### Iron (II) Magnesium (II) carbonate (MgCO3_98GPa) Crystal data

**Table:**

Mg_0.85_Fe_0.15_CO_3_	*D*_x_ = 4.614 Mg m^−^^3^
*M**_r_* = 89	Synchrotron radiation, λ = 0.2952 Å
Trigonal, *R*3*c*	Cell parameters from 65 reflections
Hall symbol: -R 3 2"c	θ = 2.7–13.9°
*a* = 4.281 (7) Å	µ = 0.25 mm^−^^1^
*c* = 12.12 (2) Å	*T* = 293 K
*V* = 192.3 (5) Å^3^	Irregular, colourless
*Z* = 6	0.01 × 0.01 × 0.01 mm
*F*(000) = 265	

### Iron (II) Magnesium (II) carbonate (MgCO3_98GPa) Data collection

**Table:**

13IDD @ APS (GSECARS) diffractometer	60 independent reflections
Radiation source: synchrotron	33 reflections with *I* > 3σ(*I*)
Synchrotron monochromator	*R*_int_ = 0.053
ω scans	θ_max_ = 15.4°, θ_min_ = 2.7°
Absorption correction: multi-scan (CrysAlisPro; Rigaku OD, 2019)	*h* = −6→6
*T*_min_ = 0.95, *T*_max_ = 1	*k* = −7→5
176 measured reflections	*l* = −18→18

### Iron (II) Magnesium (II) carbonate (MgCO3_98GPa) Refinement

**Table:**

Refinement on *F*	0 restraints
Least-squares matrix: full	1 constraint
*R*[*F*^2^ > 2σ(*F*^2^)] = 0.100	Weighting scheme based on measured s.u.'s *w* = 1/(σ^2^(*F*) + 0.000144*F*^2^)
*wR*(*F*^2^) = 0.084	(Δ/σ)_max_ < 0.001
*S* = 2.89	Δρ_max_ = 0.66 e Å^−^^3^
60 reflections	Δρ_min_ = −0.50 e Å^−^^3^
5 parameters	

### Iron (II) Magnesium (II) carbonate (MgCO3_98GPa) Fractional atomic coordinates and isotropic or equivalent isotropic displacement parameters (Å^2^)

**Table:**

	*x*	*y*	*z*	*U*_iso_*/*U*_eq_	Occ. (<1)
Mg1	0	0	0	0.0373 (13)*	0.85
Fe1	0	0	0	0.0373 (13)*	0.15
O1	0.2791 (17)	0	0.25	0.0382 (16)*	
C1	0	0	0.25	0.040 (3)*	

### Iron (II) Magnesium (II) carbonate (MgCO3_98GPa) Geometric parameters (Å, º)

**Table:**

Mg1—O1^i^	1.855 (7)	Fe1—O1^iii^	1.855 (8)
Mg1—O1^ii^	1.855 (5)	Fe1—O1^iv^	1.855 (7)
Mg1—O1^iii^	1.855 (8)	Fe1—O1^v^	1.855 (5)
Mg1—O1^iv^	1.855 (7)	Fe1—O1^vi^	1.855 (8)
Mg1—O1^v^	1.855 (5)	C1—O1	1.195 (8)
Mg1—O1^vi^	1.855 (8)	C1—O1^vii^	1.195 (8)
Fe1—O1^i^	1.855 (7)	C1—O1^viii^	1.195 (8)
Fe1—O1^ii^	1.855 (5)		
			
O1^i^—Mg1—O1^ii^	93.2 (2)	O1^i^—Fe1—O1^iv^	180
O1^i^—Mg1—O1^iii^	93.2 (2)	O1^i^—Fe1—O1^v^	86.8 (2)
O1^i^—Mg1—O1^iv^	180	O1^i^—Fe1—O1^vi^	86.8 (2)
O1^i^—Mg1—O1^v^	86.8 (2)	O1^ii^—Fe1—O1^iii^	93.2 (3)
O1^i^—Mg1—O1^vi^	86.8 (2)	O1^ii^—Fe1—O1^iv^	86.8 (2)
O1^ii^—Mg1—O1^iii^	93.2 (3)	O1^ii^—Fe1—O1^v^	180
O1^ii^—Mg1—O1^iv^	86.8 (2)	O1^ii^—Fe1—O1^vi^	86.8 (3)
O1^ii^—Mg1—O1^v^	180	O1^iii^—Fe1—O1^iv^	86.8 (2)
O1^ii^—Mg1—O1^vi^	86.8 (3)	O1^iii^—Fe1—O1^v^	86.8 (3)
O1^iii^—Mg1—O1^iv^	86.8 (2)	O1^iii^—Fe1—O1^vi^	180
O1^iii^—Mg1—O1^v^	86.8 (3)	O1^iv^—Fe1—O1^v^	93.2 (2)
O1^iii^—Mg1—O1^vi^	180	O1^iv^—Fe1—O1^vi^	93.2 (2)
O1^iv^—Mg1—O1^v^	93.2 (2)	O1^v^—Fe1—O1^vi^	93.2 (3)
O1^iv^—Mg1—O1^vi^	93.2 (2)	O1—C1—O1^vii^	120.00 (10)
O1^v^—Mg1—O1^vi^	93.2 (3)	O1—C1—O1^viii^	120.0 (5)
O1^i^—Fe1—O1^ii^	93.2 (2)	O1^vii^—C1—O1^viii^	120.0 (5)
O1^i^—Fe1—O1^iii^	93.2 (2)		

Symmetry codes: (i) *x*−2/3, *y*−1/3, *z*−1/3; (ii) −*y*+1/3, *x*−*y*−1/3, *z*−1/3; (iii) −*x*+*y*+1/3, −*x*+2/3, *z*−1/3; (iv) −*x*+2/3, −*y*+1/3, −*z*+1/3; (v) *y*−1/3, −*x*+*y*+1/3, −*z*+1/3; (vi) *x*−*y*−1/3, *x*−2/3, −*z*+1/3; (vii) −*y*, *x*−*y*, *z*; (viii) −*x*+*y*, −*x*, *z*.

### Fractional atomic coordinates and isotropic displacement parameters of (Mg_2.53_Fe_0.47_)C_3_O_9_ at 98 GPa.

**Table:**

Atom label	x	y	z	Site symmetry	U_isco_^[a]^	Occupancy
Mg1	0	0.2457 (6)	0	4g	0.0117 (13)	0.917 (17)
Fe1	0	0.2457 (6)	0	4g	0.0117 (13)	0.083 (17)
Mg2	0.1712 (7)	0	0.3146 (12)	4i	0.0086 (11)	1
Mg3	0.4441 (6)	0	0.6503 (9)	4i	0.0177 (11)	0.61 (2)
Fe3	0.4441 (6)	0	0.6503 (9)	4i	0.0177 (11)	0.39 (2)
O1	0.4097 (18)	0	0.105 (3)	4i	0.021 (2)	1
O2	0.3442 (12)	0.1683 (9)	0.4218 (18)	8j	0.0157 (15)	1
O3	0.0062 (12)	0.1898 (9)	0.2702 (19)	8j	0.0159 (17)	1
O4	0.1395 (17)	0	0.044 (3)	4i	0.020 (2)	1
O5	0.1487 (16)	0	0.575 (3)	4i	0.016 (2)	1
O6	0.2736 (13)	0.1662 (9)	0.847 (2)	8j	0.0179 (17)	1
C1	0.1347 (19)	0.1774 (13)	0.683 (3)	8j	0.017 (2)	1
C2	0.265 (3)	0	0.964 (4)	4i	0.024 (3)	1

[a] All atomic displacement parameters were refined in the isotropic approximation

### Fractional atomic coordinates and isotropic displacement parameters of (Mg_0.85_Fe_0.15_)CO_3_ at 98 GPa.

**Table:**

Atom label	x	y	z	Site symmetry	U_isco_^[a]^	Occupancy
Mg1	0	0	0	6b	0.0373 (13)	0.85
Fe1	0	0	0	6b	0.0373 (13)	0.15
O1	0.2791 (17)	0	0.25	18e	0.0382 (16)	1
C1	0	0	0.25	6a	0.04 (3)	1

[a] All atomic displacement parameters were refined in the isotropic approximation
